# Associations between maternal microbiome, metabolome and incidence of low-birth weight in Guatemalan participants from the Women First Trial

**DOI:** 10.3389/fmicb.2024.1456087

**Published:** 2024-10-15

**Authors:** Meghan L. Ruebel, Stephanie P. Gilley, Laxmi Yeruva, Minghua Tang, Daniel N. Frank, Ana Garcés, Lester Figueroa, Renny S. Lan, Hailemariam Abrha Assress, Jennifer F. Kemp, Jamie L. E. Westcott, K. Michael Hambidge, Kartik Shankar, Nancy F. Krebs

**Affiliations:** ^1^Microbiome and Metabolism Research Unit, USDA-ARS, Southeast Area USDA-ARS, Little Rock, AR, United States; ^2^Arkansas Children's Nutrition Center, Little Rock, AR, United States; ^3^Department of Pediatrics, Section of Nutrition, University of Colorado School of Medicine, Aurora, CO, United States; ^4^Department of Medicine, Division of Infectious Disease, University of Colorado School of Medicine, Aurora, CO, United States; ^5^Maternal Infant Health Center, Instituto de Nutrición de Centro América y Panamá (INCAP), Guatemala City, Guatemala; ^6^Department of Pediatrics, Section of Developmental Nutrition, University of Arkansas for Medical Sciences, Little Rock, AR, United States

**Keywords:** microbiome, pregnancy, growth, low birth weight, preconception

## Abstract

**Background:**

Low birth weight (LBW; <2,500 g) affects approximately 15 to 20 percent of global births annually and is associated with suboptimal child development. Recent studies suggest a link between the maternal gut microbiome and poor obstetric and perinatal outcomes. The goal of this study was to examine relationships between maternal microbial taxa, fecal metabolites, and maternal anthropometry on incidence of LBW in resource-limited settings.

**Methods:**

This was a secondary analysis of the Women First trial conducted in a semi-rural region of Guatemala. Maternal weight was measured at 12 and 34 weeks (wk) of gestation. Infant anthropometry measures were collected within 48 h of delivery. Maternal fecal samples at 12 and 34 weeks were used for microbiome (16S rRNA gene amplicon sequencing) and metabolomics analysis (34 wk). Linear mixed models using the MaAslin2 package were utilized to assess changes in microbiome associated with LBW. Predictive models using gradient boosted machines (XGBoost) were developed using the H2o.ai engine.

**Results:**

No differences in β-diversity were observed at either time point between mothers with LBW infants relative to normal weight (NW) infants. Simpson diversity at 12 and 34 weeks was lower in mothers with LBW infants. Notable differences in genus-level abundance between LBW and NW mothers (*p* < 0.05) were observed at 12 weeks with increasing abundances of *Barnesiella*, *Faecalibacterium*, *Sutterella*, and *Bacterioides*. At 34 weeks, there were lower abundances of *Magasphaera*, *Phascolarctobacterium*, and *Turicibacter* and higher abundances of *Bacteriodes*, and *Fusobacterium* in mothers with LBW infants. Fecal metabolites related to bile acids, tryptophan metabolism and fatty acid related metabolites changed in mothers with LBW infants. Classification models to predict LBW based on maternal anthropometry and predicted microbial functions showed moderate performance.

**Conclusion:**

Collectively, the findings indicate that alterations in the maternal microbiome and metabolome were associated with LBW. Future research should target functional and predictive roles of the maternal gut microbiome in infant birth outcomes including birthweight.

## 1 Introduction

Globally, more than 20 million infants annually are born with low-birth weight (LBW), defined as a birthweight <2,500 grams. The global burden of LBW is disproportionate with ~95% of LBW infants being born in low-and middle-income countries (Cutland et al., 2017; Bramer, 1988). While, the prevalence of LBW is ~8.5% in the United States (Burris and Hacker, 2017; Blencowe et al., 2019), LBW infants are twice as likely to be stunted in childhood and is one of the leading causes of diminished child development (Vats et al., 2024). Thus reducing LBW incidence by 30% by 2025 is a prioritiy of the Sustainable Development Goals as well as part of the six global nutrition targets by the World Health Assembly (McGuire, 2015). Recent work from over 148 countries suggests that this will be a daunting task to meet, as it will require more than double the current annual reduction rate (Blencowe et al., 2019).

LBW is associated with a variety of maternal factors including age, infection, high blood pressure, nutritional status, and environmental exposures (Victora et al., 2008; Cluzeni et al., 2023). Of these factors, maternal nutrition, specifically adequacy of minerals and micronutrients, is thought to be a significant contributor to *in utero* growth and long-term adulthood disease risk (Victora et al., 2008; Tranidou et al., 2024; Freire et al., 2024). Emerging evidence has also suggests that the maternal gut microbiome during pregnancy can impact obstetric and perinatal outcomes, such as birthweight (Gough et al., 2021; Vinturache et al., 2016). A recent study conducted in Zimbabwe, part of the SHINE trial, showed that fecal microbiome from pregnant women could predict infant birth weight and neonatal growth outcomes (Gough et al., 2021). Moreover, microbial metabolites present in the gut and systemic circulation, are thought to be an important component of host-microbiome communication. Research by Tang et al. showed evidence that fecal metabolites in pregnant women are associated with neonatal growth outcomes, specifically fetal growth restriction (Tang et al., 2024). However, no studies to-date have examined the linked effects of maternal malnutrition and abundance of microbial taxa and/or fecal metabolites during pregnancy on the risk of LBW.

We hypothesized that maternal microbiome and metabolites in pregnancy will be associated with a LBW compared to normal weight in infants. To address this question we utilized samples from Guatemala women that tend to have shorter height, one of the 4 sites of the Women First (WF): Preconception Maternal Intervention Nutrition Trial (Arriaza et al., 2022) with the high rates of LBW (~15% of all births) and stunting in children by 2 years of age (~66%; Krebs et al., 2022). We leveraged maternal fecal samples during pregnancy to examine relationships between maternal microbiome (taxonomic abundance), fecal metabolites, and maternal anthropometry on incidence of LBW. In addition, machine learning models were implemented to determine whether microbial taxonomic abundance and other maternal variables are predictive of neonatal LBW.

## 2 Methods

### 2.1 Study design and participants

**Figure 1:**
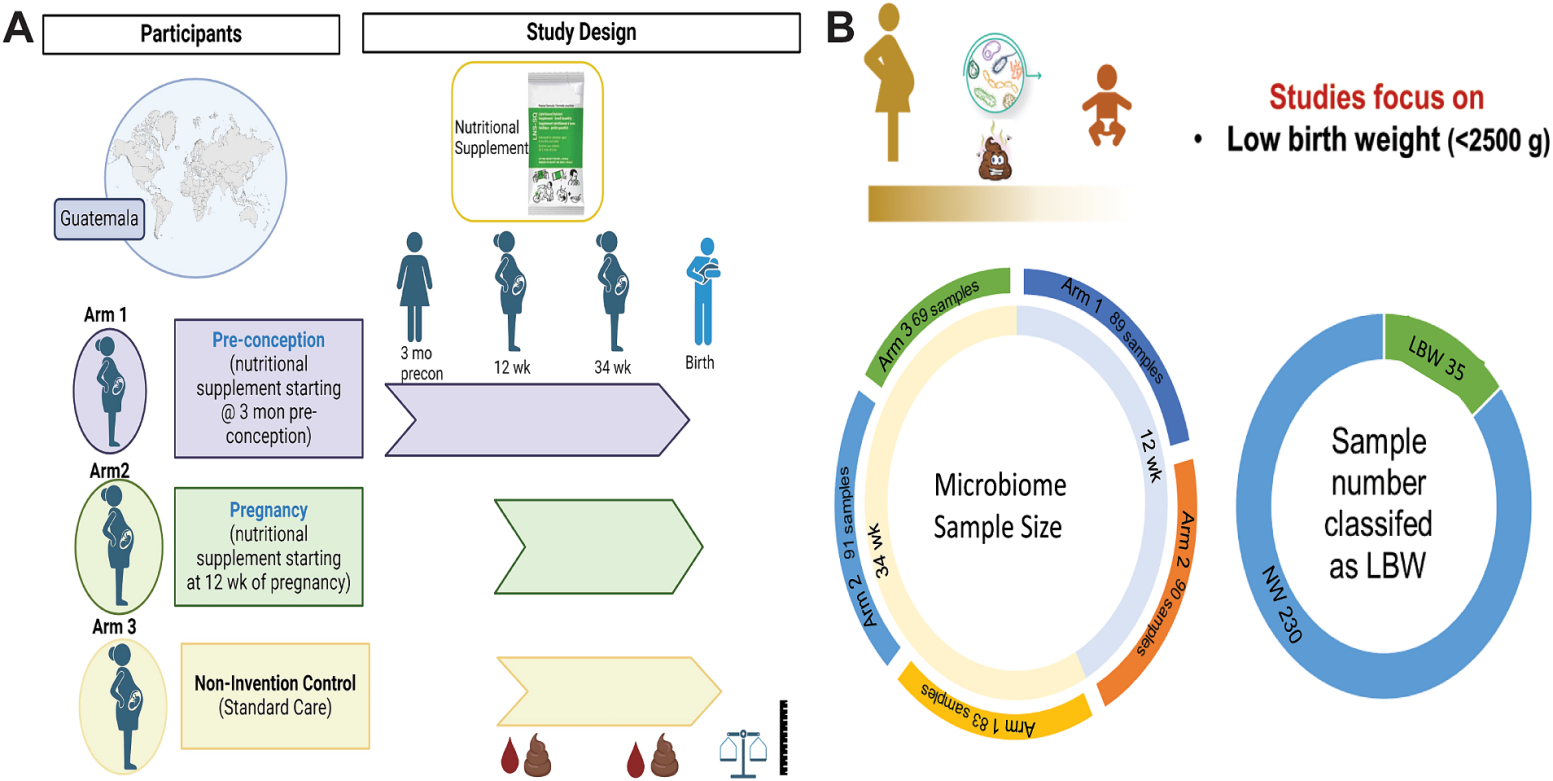
Women first trial experimental design and sample size. **(A)** Experimental design of Women First Trial and treatment arms. **(B)** Sample size of microbiome samples included in this analysis by timepoint and treatment arms. As well as sample number of low birth weight (LBW) infants compared to normal weight (NW) infants included in the study. Created with BioRender.com

This is a secondary analysis of the Women First: Preconception Maternal Intervention Nutrition Trial (ClinicalTrials.gov ID: NCT01883193), that included women of reproductive age from four resource-limited countries and was focused on improving infant outcomes such as birth weight and length (Hambidge et al., 2019). This trial was unique in testing the effects of a combination of macro-and micronutrients on maternal–infant outcomes and included multiple biospecimens, including stool samples. For this analysis, only participants recruited from Chimaltenango, Guatemala were included. The full study details can be found in previous publications (Hambidge et al., 2019; Tang et al., 2022). Briefly, participants were randomized into three different treatment arms: Arm 1 received a daily small-quantity lipid-based micronutrient supplement (sqLNS) starting at ≥3 months before conception and throughout pregnancy, Arm 2 received daily sqLNS supplementation starting at 12 weeks of gestation and through the remainder of pregnancy, and Arm 3 received only the local standard of care which included iron and folate supplementation (Figure 1A).

All participants recruited for this study were between 16 and 35 years old, parity 0–5, and planned to conceive during the following 18 months. Written informed consent was obtained from all the participants. The study protocol was approved by Institutional Review Board at the University of Colorado and Comité de Ética de la Universidad Francisco Marroquín.

### 2.2 Maternal and infant anthropometrics

Maternal weight and height were obtained by trained study personnel and measured at enrollment. Neonatal birth length, birth weight, and head circumference were collected within 24 h of delivery by trained research staff who were blinded to treatment arms. All measurements were taken in triplicates using neonatal stadiometers (Ellard Instrumentation Ltd., Monroe WA), Seca 334 electronic scales, and Seca 201 measuring tapes (Seca North America, Chino CA). First-trimester ultrasound data were used to determine gestational age. Birth weight, length, and head circumference measurements were obtained and transformed to z-scores adjusted for gestational age using INTERGROWTH-21^st^ standards (https://intergrowth21.tghn.org/; Papageorghiou et al., 2014).

### 2.3 Fecal sample collection

Duplicate fecal samples were collected at two timepoints during pregnancy: 12 weeks (Arm 1 taking sqLNS, and Arm 2 before starting sqLNS) and 34 weeks gestation (Arms 1, 2, and 3). Stool was collected into fecal bags using a sterile scoop and placed into a Styrofoam container with ice or ice packs. The research team picked up samples the day of stool passage, transferred samples back to facility where they were aliquoted into storage tubes with and without 3 ml of RNAlater™ and frozen at −80°C. The RNALater™ aliquot was used for microbiome analysis and the other aliquot was used for metabolomics. Samples were shipped to the University of Colorado Pediatric Nutrition Laboratory and stored at −80°C until analyses.

### 2.4 16S rRNA sequencing and analysis

Generation and pre-processing of the 16S rRNA gene sequence datasets analyzed herein was previously reported in Tang et al., 2022; sequence data and associated clinical/demographic metadata are available through the NCBI sequence read archive (Bioproject PRJNA553183). In Supplementary Table 1, sample identifiers from this BioProject and relevant metadata (eg. LBW status) are provided. Microbial sequence counts, taxonomy information and sample metadata were imported into the phyloseq package (McMurdie and Holmes, 2013). The microbiome package ‘core’ function was used to eliminate taxa that did not have at least 5 counts in 5% of samples. Alpha diversity was determined using the microeco package (Liu et al., 2021) and Student’s t-test were used to test differences between groups (LBW vs. NW). Beta diversity was assessed using Bray–Curtis dissimilarity. Multidimensional scaling was used to visualize beta diversity for each group and statistical difference was tested using PERMANOVA with 999 permutations. Multivariable associations between LBW status and taxonomic abundance were assessed using the MaAsLin2 package (Mallick et al., 2021). For normalization of the data we used MaAsLin2 default settings, which included total sum scaling (TSS) and log transformation. LBW and treatment arm were considered fixed effects, and analyses were adjusted for regional clusters, sex, maternal age, and sample batch. Taxa were agglomerated at the genus level. All nominal *p*-values at *p* < 0.05 were considered significant. The relative abundance of taxa was visualized on a log-transformed axis in figures.

### 2.5 Fecal metabolomics and analysis

Approximately 100 mg of frozen stool at 34 weeks from LBW and NW groups (*n* = 20 per group), were extracted and subjected to untargeted metabolomics analyses using liquid chromatography/mass spectrometry (LC–MS) at the Metabolomics and Analytical Chemistry Core-Arkansas Children’s Nutrition Center. Briefly, 500 μl of LC–MS grade 50% of MeOH in water and 1 ml of Acetonitrile was added to the stool. The mixture was quantitatively transferred to screw cap tube containing 200 μl of 1.44 mm beads, 100 μl of 0.5 mm beads and 3 beads of 2.8 mm beads. Samples were homogenized at 5,300 rpm with a Precellys 24 for two 30 s cycles. The mixture was vortexed for 10 min at 4°C on a ThermoMixer (Eppendorf Inc., Enfield, CT) and then centrifuged at 4,347 g at 4°C for 10 min. 700 μl of the supernatant was aliquoted and subsequently dried by using a vacuum concentrator (SpeedVac SPD210, Thermo Fisher Scientific Waltham, MA). Extracts were then reconstituted in 250 μl of 5% methanol spiked with 1,000 ng ml^−1^ sulfadimethoxine (SDMO) for immediate analysis. Instrumental pooled quality control (QC) samples were prepared by pooling equal volumes of each sample extract (50 μl). Chromatographic separations were conducted on a Dionex Ultimate 3,000 UHPLC with a Premier CSH C18 reversed phase column (2.1 × 100 mm, 1.7 μm). An Orbitrap Exploris 480 mass spectrometer (ThermoFisher Scientific, Waltham, MA) interfaced with the Vanquish UHPLC system and fitted with heat-electrospray ionization (HESI) probes was used for instrumental analysis. Detailed information about chromatographic and mass spectrometry conditions is provided in the Supplementary methods. Using an untargeted metabolomics workflow, the acquired data (full MS and data dependent MS2) was processed using Compound Discoverer 3.3, which is described in detail in the Supplementary methods. Peak intensities of resulting metabolite features with Level 2 identification were utilized for statistical analysis. Data pre-processing included normalizations to exact sample (stool) weights, log transformation, and auto-scaling implemented in the MetaboAnalyst package. Z-scores of metabolite abundance were further utilized in linear regression models conducted in R. These models adjusted for recruitment cluster and treatment arm. Significance was set using an FDR *p* < 0.05.

### 2.6 Predictive machine-learning model

For predictive machine-learning modelling, we used LBW as the outcome variable (categorical) and taxa abundance (z-scores) and maternal anthropometry as predictive features. We used only the 34-week samples in this analysis, which included a total of 243 subjects that were randomly split into training (80% of samples) and test datasets (20% of samples) using the createDataPartition function from the caret package. Model development and validation were done in R using the H2o.ai engine and the h2o package (Xiao et al., 2024; LeDell and Poirer, 2020). Initial model evaluation was conducted using the AutoML function in H2o and employed multiple model families, including distributed random forest, GLM (generalized linear model), gradient boosted machines (GBM, including XGBoost). The process of hyperparameter tuning and grid search were performed using AutoML (LeDell and Poirer, 2020). Initial results revealed that XGBoost models had the best performance, and hence were chosen for further refinement. XGBoost is a supervised learning algorithm forward-learning process called boosting to yield accurate models. Model performance was evaluated by the following measures of accuracy, sensitivity, specificity, mean per-class error, and precision derived from the confusion matrix. Model performance was evaluated by training on 80% of the samples and testing the remaining 20%. Important features contributing to the model were derived using a scaled variable importance (VIPs scores) determined by calculating the relative influence of each variable.

### 2.7 Statistical analysis

All other statistical analyses were performed in GraphPad Prism version 10.1.1. Data are presented as mean ± SD or percentages. Statistical significance of *p* < 0.05 was calculated using Student t-test or chi-squared tests.

## 3 Results

### 3.1 Participant characteristics

Microbiome samples from a total of 265 mother-infant pairs were used in this study. Of those 179 were 12-week samples (Arm 1: 89; Arm 2: 90) and 243 were 34-week samples (Arm 1: 83, Arm 2: 91; Arm 3: 69). Maternal–infant data were grouped based on birth weight of infants and classified as LBW (*n* = 35, 15%) or normal weight (NW, *n* = 230; Figure 1B). Maternal body mass index (BMI) at both 12 and 34 weeks was significantly higher in the mothers that had NW infants (Table 1). There were no differences in maternal age, parity, mode of delivery, antibiotic use, or infant sex in between birth weight groups (Table 1). It is also important to note that on average ~ 75% of women in this study cohort gave birth vaginally. Infants in the LBW group had significantly higher rates of being classified as small for gestational age (SGA) and lower gestational-age adjusted z-scores for birth weight-for-age (WAZ), length-for-age (LAZ), and head circumference-for-age (HCAZ) compared to NW weight infants (Table 1).

**Table 1:** Participant characteristics by time and birth outcome.

	12 weeks			34 weeks		
Maternal outcomes	LBW Infant	NW Infant	*p*-value	LBW Infant	NW Infant	*p*-value
Age (y)	23.6 ± 4.1	22.9 ± 4.0	0.57	23.0 ± 3.8	23.8 ± 3.9	0.39
BMI (kg/m^2^)	23.5 ± 4.3	25.6 ± 4.3	0.02	23.7 ± 4.0	25.7 ± 4.1	**0.009**
Underweight (%)	7.1	0.0	0.001	3.0	0.0	**0.01**
Normal weight (%)	57.1	50.0	0.49	63.6	46.9	0.07
Overweight/obese (%)	35.7	50.0	0.16	33.3	53.1	**0.03**
Parity at enrollment	1.5 ± 1.2	1.8 ± 1.1	0.13	1.6 ± 1.2	1.9 ± 1.1	0.28
Mode of delivery (% vaginal)	78.6	68.9	0.3	81.8	69.0	0.13
Antibiotic use (% yes)	0.0	0.9	0.72	0.0	0.5	0.69
Infant outcomes						
Infant sex (% female)	39.3	47.7	0.41	42.4	46.7	0.65
SGA classification (% yes)	88.0	12.0	<0.0001	89.7	10.3	**<0.0001**
Infant weight at 24H (kg)	2.26 ± 0.17	2.99 ± 0.30	<0.0001	2.27 ± 0.16	3.01 ± 0.29	**<0.0001**
Infant height at 24 H (cm)	44.96 ± 1.69	48.13 ± 1.49	<0.0001	44.99 ± 1.58	48.06 ± 1.54	**<0.0001**
Infant head circumference at 24H (cm)	37.9 ± 1.2	39.3 ± 1.1	<0.0001	37.9 ± 1.2	39.3 ± 1.2	**<0.0001**
Gestational Age Adjusted LAZ	−1.84 ± 0.77	−0.56 ± 0.76	<0.0001	−1.86 ± 0.73	−0.64 ± 0.76	**<0.0001**
Gestational Age Adjusted WAZ	−1.85 ± 0.50	−0.55 ± 0.72	<0.0001	−1.86 ± 0.46	−0.54 ± 0.70	**<0.0001**
Gestational Age Adjusted HCAZ	−1.40 ± 0.85	−0.06 ± 0.91	<0.0001	−1.37 ± 0.79	−0.11 ± 0.91	**<0.0001**

Data presented as mean ± SD or percentages. Statistical significance of *p* < 0.05 was calculated using student *t*-test or chi-squared tests. Bold signifies *p* < 0.05.

### 3.2 Fecal microbiome composition of pregnant mothers differed based on LBW vs. NW infants’ status

**Figure 2:**
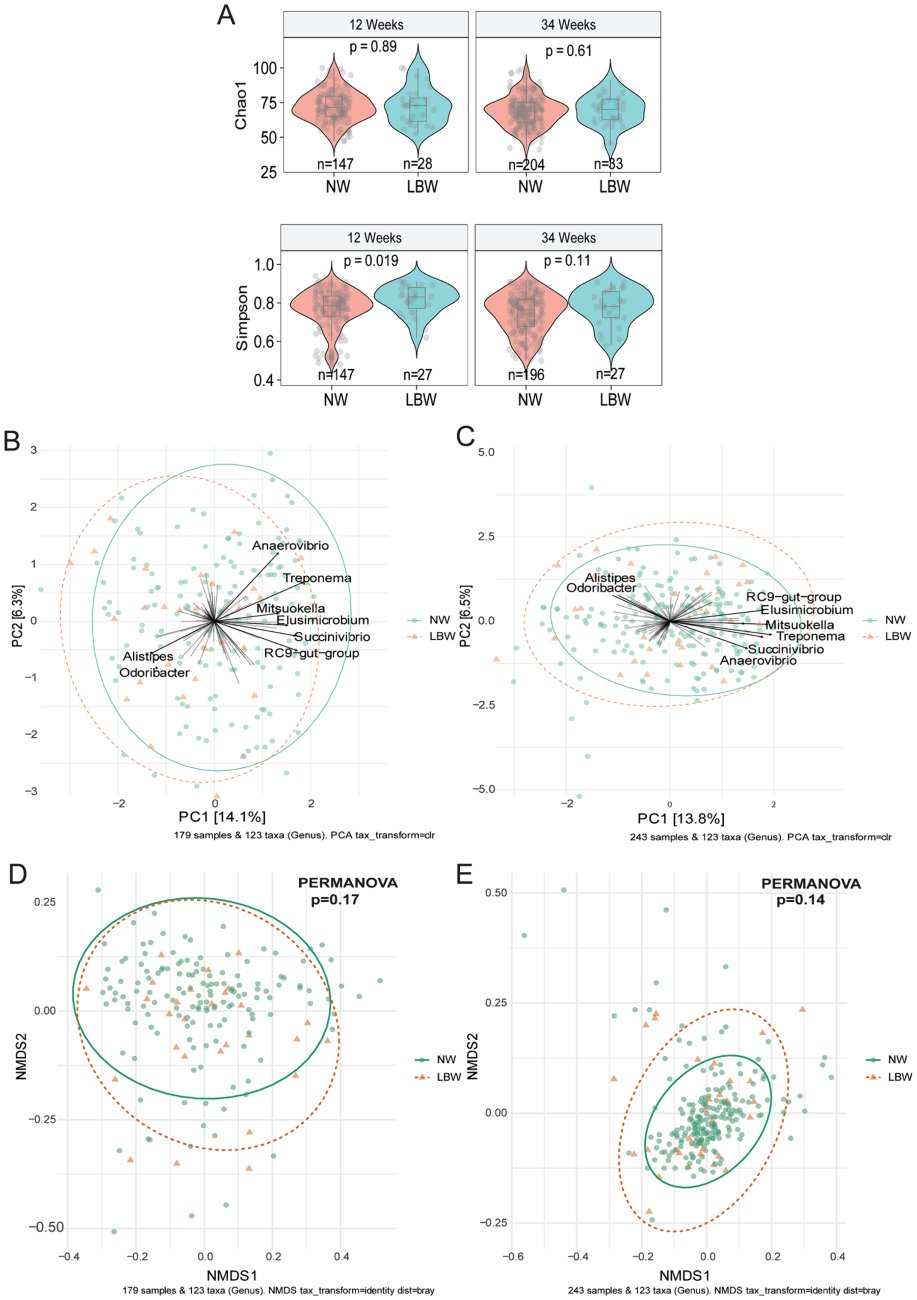
Low birth weight (LBW) status and gut microbiome composition at 12 and 34 weeks of gestation. **(A)** Violin plots of alpha diversity indices associated with LBW status at 12-and 34-week gestation. All main effects of LBW were *p* < 0.05. Pairwise *p*-values were derived using Wilcoxon test. **(B)** Bi-plot representation of principal components analysis of genus-level taxa at 12 and **(C)** 34 weeks of gestation by LBW status. **(D)** Non-metric dimensional scaling (NMDS) ordination of Bray-Curtis similarities of samples at 12 and **(E)** 34 weeks by LBW status.

To examine changes in the maternal gut microbiome associated with LBW, we evaluated maternal fecal microbiome at 12 weeks (Trimester 1) and 34 weeks (Trimester 3). Alpha diversity only differed at 12 weeks by one of 6 (Observed, Chao1, ACE, Shannon, Simpson, and Fischer) indices assessed. Mothers with LBW infants had significantly higher evenness (Simpson) at 12 weeks compared to NW (Figure 2A). There was no changes in alpha diversity parameters at 34 weeks of gestation. Non-metric dimensional scaling (NMDS) ordination of Bray Curtis dissimilarity and unsupervised Principal Component Analysis (PCA) plots showed no significant differences by PERMANOVA in global genus-level composition (beta diversity) due to LBW at either 12 or 34 weeks of gestation (Figures 2B–E). We also analyzed β-diversity comparing intervention arms and found no difference in intervention arm at either timepoint (data not shown).

**Figure 3:**
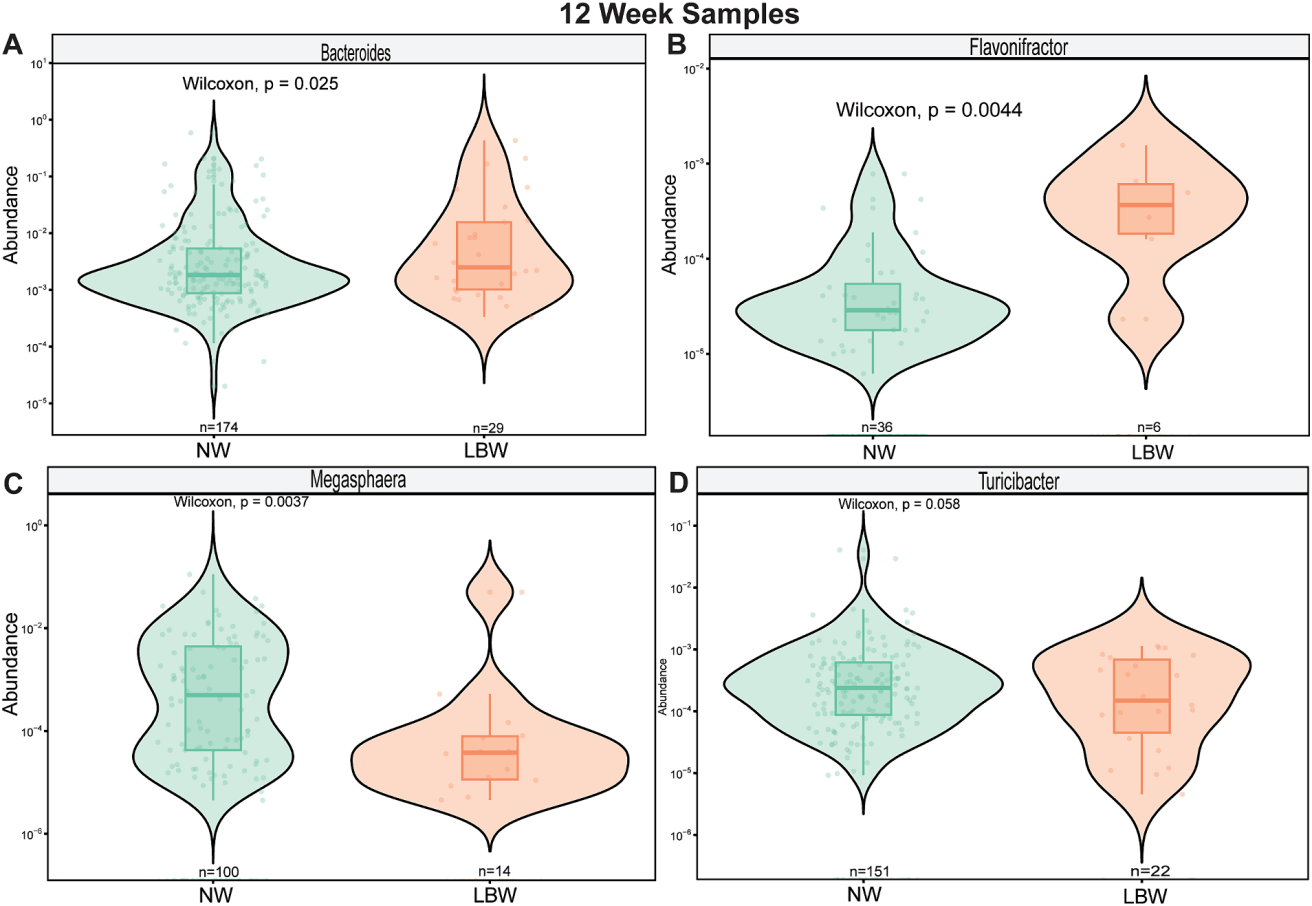
Violin plots showing levels of differentially expressed genus-level taxa by LBW status at 12 weeks of gestation. **(A)** *Bacteroides*, **(B)** *Barnesiella* **(C)** *Faecalibacterium*, and **(D)** *Sutterella*. Differential abundance was assessed using MaAsLin2. All main effects of LBW were *p* < 0.05. Pairwise *p*-values were derived using Wilcoxon test.

**Figure 4:**
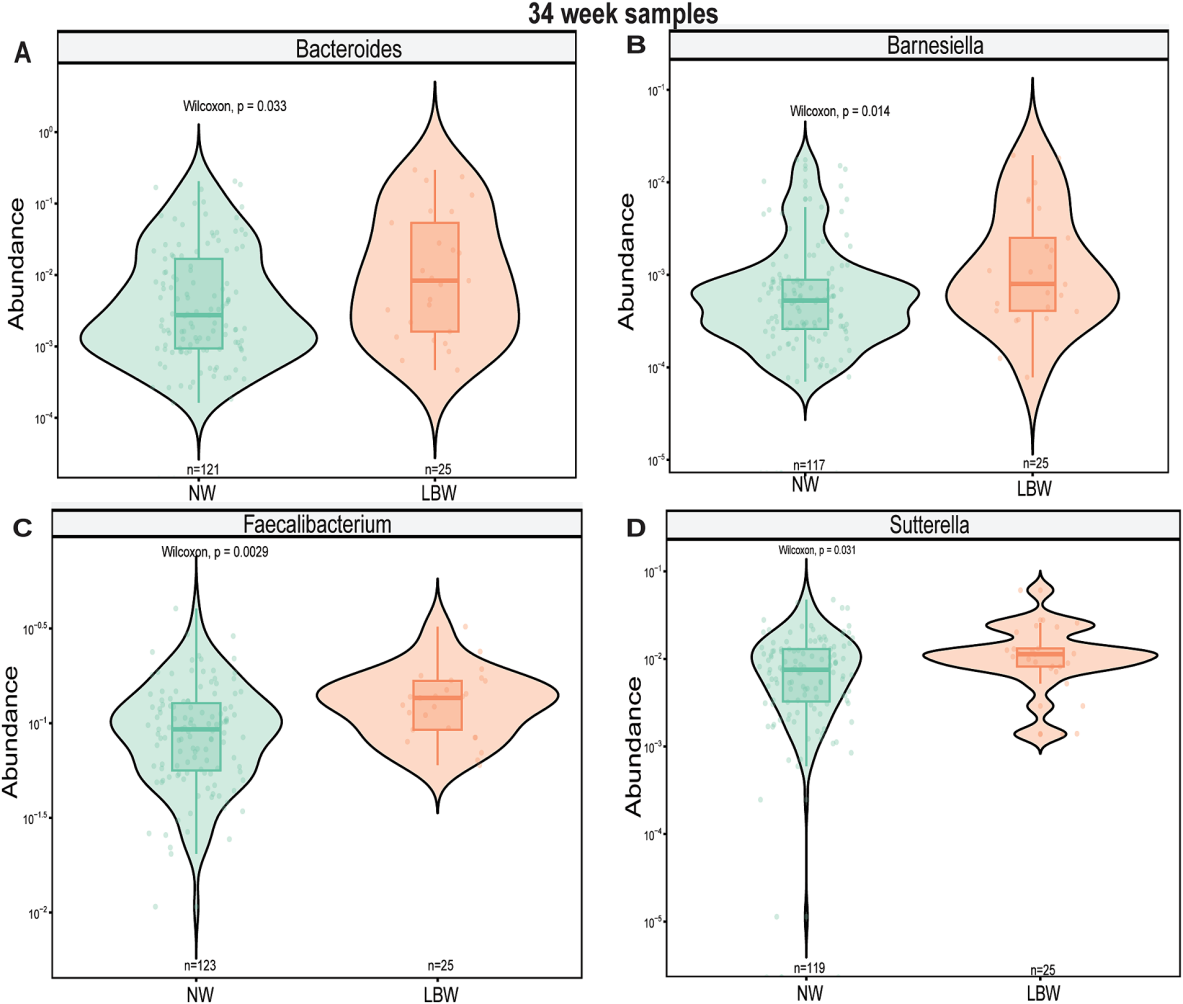
Violin plots showing levels of differentially expressed genus-level taxa by low bith weight (LBW) status at 34 weeks of gestation. **(A)** *Bacteroides*, **(B)** *Flavonifactor*, **(C)** *Megasphera*, and **(D)** *Turicibacter*. Differential abundance was assessed using MaAsLin2. All main effects of LBW were *p* < 0.05. Pairwise *p*-values were derived using Wilcoxon test.

Next, we examined changes in genus-level bacterial abundance in both the 12-and 34-week samples associated with LBW. At 12 weeks, we identified significantly increased abundance of *Barnesiella*, *Faecalibacterium*, *Sutterella*, *Odoribacter*, *Hafnia*, and *Bacterioides* in the fecal microbiome of women who had LBW infants compared to NW infants (Figure 3, Table 2). During late pregnancy (34 weeks), mothers of LBW infants had significantly lower abundance of *Megasphaera*, *Phascolarctobacterium*, and *Turicibacter* and increased abundance of *Bacteriodes*, *Flavonifractor*, *Acinetobacter*, and *Fusobacterium* compared to NW infants (Figure 4; Table 2).

**Table 2:** Fecal Microbial Abundances at Genus Level from Pregnant Women in Guatemala with low birth weight (LBW) infants compared to normal weight (NW) infants at both 12 and 34 weeks of Gestation.

Fecal samples from pregnant women who had LBW compared normal weight infants						
Phylum	Family	Genus	Coefficient	Std. Dev.	*p* value	Q value
12 weeks gestation						
Bacteroidetes	*Porphyromonadaceae*	*Barnesiella*	0.403	0.13	**0.002**	0.17
Firmicutes	*Ruminococcaceae*	*Faecalibacterium*	0.171	0.06	**0.003**	0.17
Proteobacteria	*Alcaligenaceae*	*Sutterella*	0.384	0.15	**0.012**	0.37
Bacteroidetes	*Porphyromonadaceae*	*Odoribacter*	0.599	0.25	**0.016**	0.37
Proteobacteria	*Enterobacteriaceae*	*Hafnia*	0.066	0.03	**0.029**	0.49
Bacteroidetes	*Bacteroidaceae*	*Bacteroides*	0.363	0.18	**0.042**	0.53
Proteobacteria	*Alcaligenaceae*	*Parasutterella*	0.409	0.21	0.055	0.61
Firmicutes	*Lachnospiraceae*	*Lachnospira*	0.285	0.15	0.059	0.61
Bacteroidetes	*Prevotellaceae*	*Paraprevotella*	0.425	0.23	0.070	0.62
Firmicutes	*Erysipelotrichaceae*	*Solobacterium*	−0.447	0.25	0.078	0.62
Bacteroidetes	*Prevotellaceae*	*Prevotella*	−0.205	0.12	0.085	0.62
Firmicutes	*Lachnospiraceae*	*Roseburia*	0.160	0.09	0.093	0.63
34 weeks gestation						
Firmicutes	*Veillonellaceae*	*Megasphaera*	−0.636	0.27	**0.020**	0.97
Firmicutes	*Acidaminococcaceae*	*Phascolarctobacterium*	−0.450	0.22	**0.025**	0.97
Bacteroidetes	*Bacteroidaceae*	*Bacteroides*	0.313	0.14	**0.025**	0.97
Firmicutes	*Ruminococcaceae*	*Flavonifractor*	0.215	0.10	**0.033**	0.97
Proteobacteria	*Moraxellaceae*	*Acinetobacter*	0.127	0.06	**0.034**	0.97
Fusobacteria	*Fusobacteriaceae*	*Fusobacterium*	0.294	0.14	**0.042**	0.97
Firmicutes	*Erysipelotrichaceae*	*Turicibacter*	−0.326	0.17	0.053	0.97
Proteobacteria	*Enterobacteriaceae*	*Escherichia-Shigella*	−0.284	0.16	0.081	0.97

Statistical differences between groups were determined by MaAsLin2, adjusting for regional clusters, sex, maternal age, and sample batch. Significance was set at *p* < 0.05, using nominal p-values. Bold signifies *p* < 0.05.

### 3.3 Prediction of LBW using microbiome data

**Figure 5:**
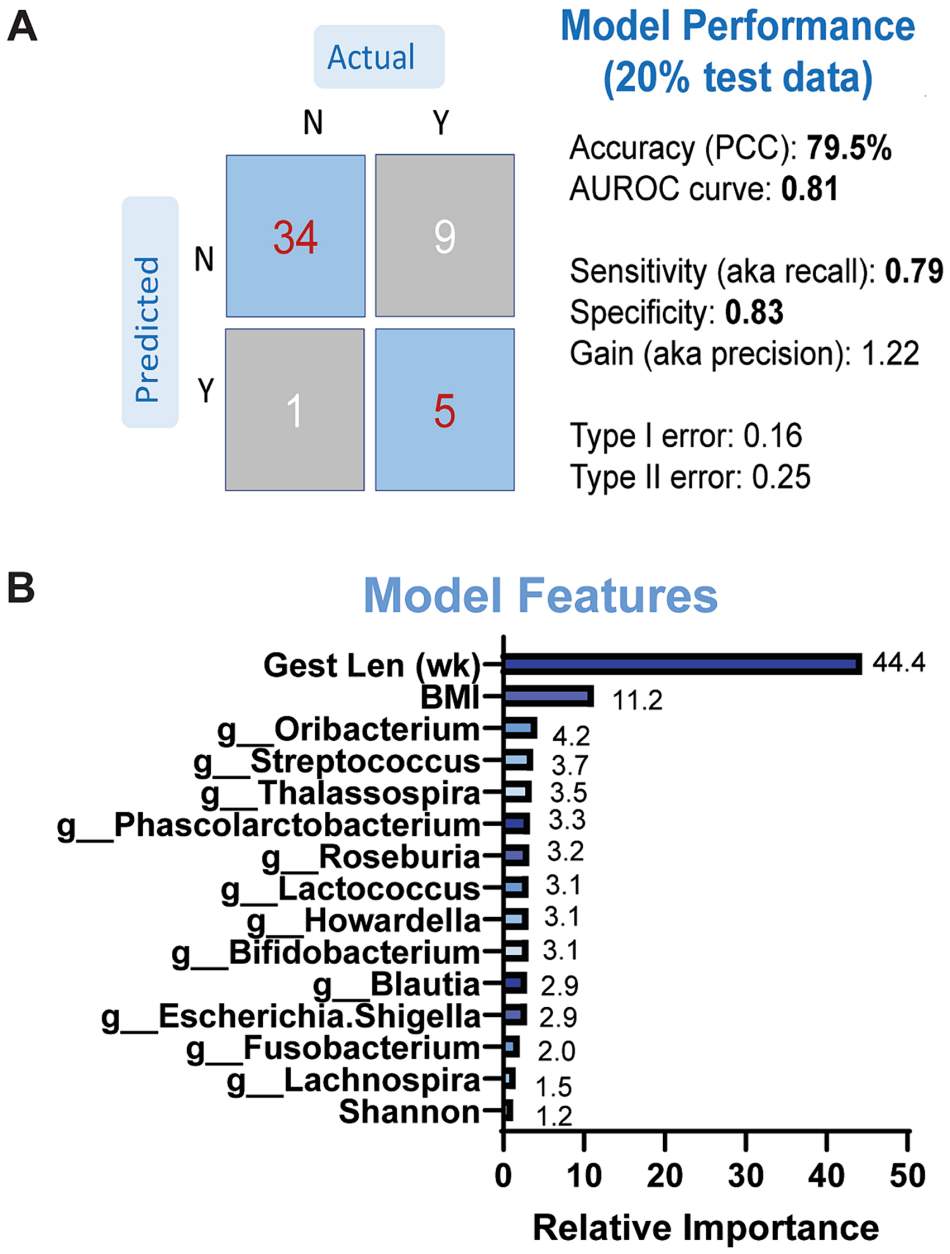
Results of GBM models predicting LBW status based on maternal microbiome and anthropometry. **(A)** Model performance was evaluated on separate (20%) dataset. **(B)** Model features by relative importance that show predictable of LBW status.

We then determined whether microbial taxonomic abundance and other maternal variables were predictive of neonatal LBW using a gradient-boosted machine-learning model. Due to the small sample size that was used to conduct fecal metabolomics, we did not include metabolite data into our model. We first evaluated the performance and overall accuracy of our model. Our model showed moderate to good performance, with an overall accuracy of 79.5%, sensitivity at 0.79 and specificity at 0.83 (Figure 5A). The top features contributing to model prediction based on relative importance were maternal BMI, gestational length, and genus-level taxonomic abundance of *Oribacterium*, *Streptococcus*, and Thalassospiria (Figure 5B). We repeated the same models excluding the microbiome data but retaining the maternal variables. The performance of the models was classified as poor with less than 60% accuracy (data not shown), suggesting that maternal fecal microbiome has an important contribution on model performance and the ability to predict LBW status in infants.

### 3.4 Maternal fecal metabolomics

**Figure 6:**
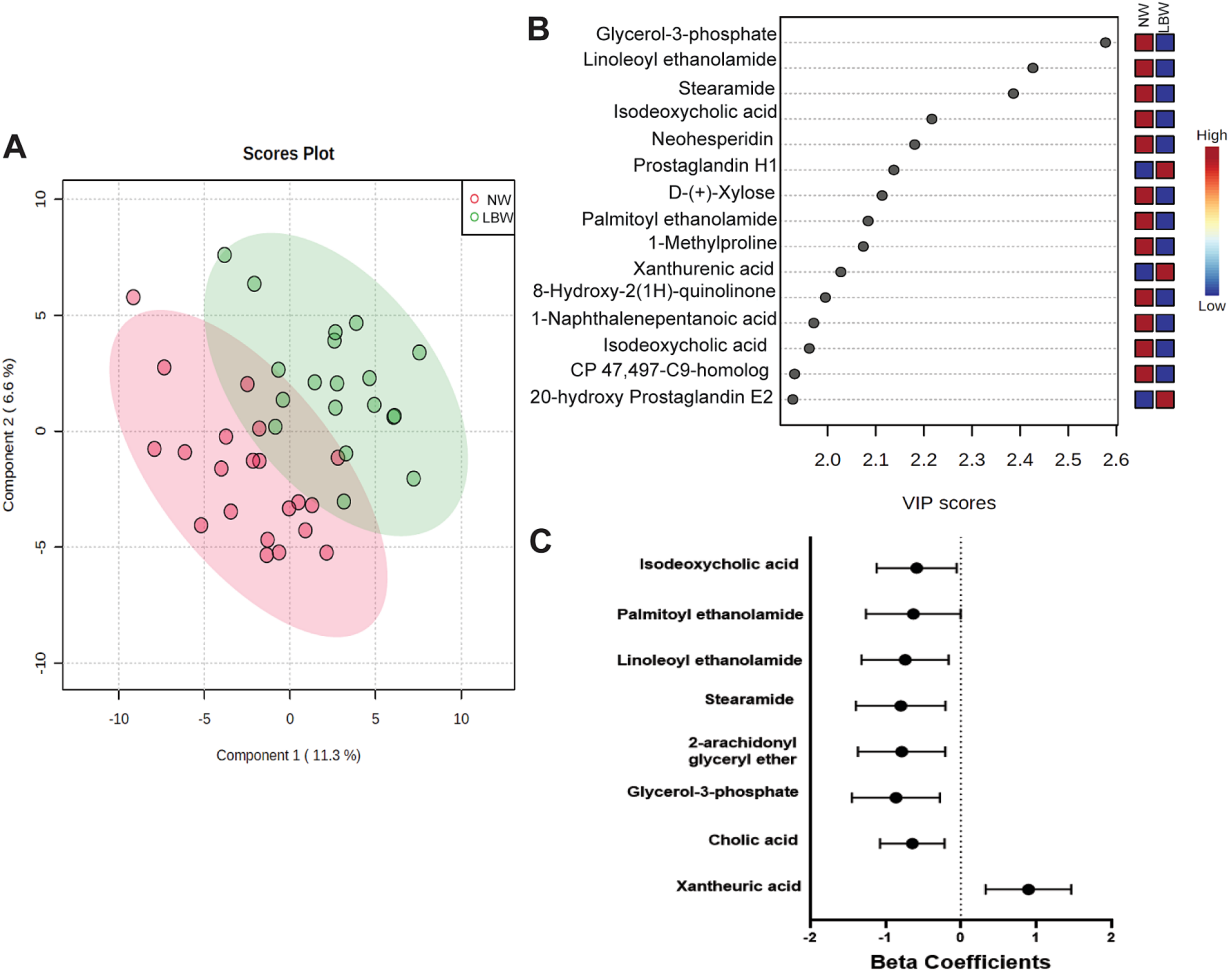
Results of untargeted metabolomics of stool samples at 34 weeks of pregnancy by low birth weight (LBW) status. Samples were analyzed via LC–MS/MS. Sample size by LBW status: LBW (*n* = 20) and normal weight (NW; *n* = 20). Analysis conducted in Metaboanaylst included **(A)** 2-D loading plot of partial least squares discriminant analysis (PLDS-DA) with selected metabolites using average variable importance in projection (VIP) scores by LBW vs. NW infants. **(B)** Selected metabolites identified with a VIP score of 2.0 but not significant, FDR *p* > 0.05. **(C)** Only metabolites with high confidence of annotation were further analyzed by using linear regression against LBW controlling for treatment arm and regional clusters. Graph depicts beta-coefficients and 95% CI for a subset of metabolites showing significant associations from linear regression model (*p*-value <0.05).

A subset of fecal samples at 34-week gestation (*n* = 20, LBW and *n* = 20, NW) underwent untargeted metabolomic analysis to test whether certain metabolites were associated with having a LBW infant. We identified 109 metabolites in positive mode and 125 metabolites in negative mode at Level 2 annotation. PCA did not show separation between fecal metabolome from mothers that had LBW infants compared to those with NW infants (data not shown). However, partial least squares discriminant analysis (PLDS-DA) with selected metabolites, that were identified using average VIP scores, showed separation between samples from LBW vs. NW infants (Figure 6A). Although no metabolites met the pre-defined FDR cut-off (*p* < 0.05), a few had VIP scores greater than 2 (Figure 6B), likely due to the smaller sample size per group. Next, we used z-scores of metabolite abundance to determine if there were fecal metabolites that differed in the mothers who had LBW or NW infants. Specifically, we used linear regression that was adjusted for recruitment cluster and treatment arm. We identified 26 metabolites that were significantly different between LBW and NW infants, 2 metabolites (Xanthurenic acid and pi-Methylimidazoleacetic acid) had increased abundance, and 24 metabolites were decreased in mothers that had LBW infants (adjusted p < 0.05). These included bile acids (Cholic acid, Isodeoxycholic acid), fatty acids (Stearamide, Glycerol-3-phosphate, Eicosapentaenoic acid methyl ester) and other metabolites related to endocannabinoids [(9Z)-9-Octadecenamide, 2-arachidonyl glyceryl ether, Linoleoyl ethanolamide, and Palmitoyl ethanolamide; Figure 6C; Table 3].

**Table 3:** Fecal metabolites of pregnant mothers at 34-week gestation with low birth weight (LBW; *N* = 20) compared to normal weight (NW; *N* = 20) infants.

Metabolites	Coefficient	SE	CI_low	CI_high	*p*-value
Stearamide	−0.964	0.288	−1.548	−0.380	0.002
Cholic acid	−0.716	0.215	−1.154	−0.279	0.002
Xanthurenic acid	0.952	0.292	0.358	1.546	0.003
(9Z)-9-Octadecenamide	−0.845	0.270	−1.393	−0.296	0.004
Cholic acid	−0.540	0.175	−0.898	−0.182	0.004
2-arachidonyl glyceryl ether	−0.774	0.282	−1.349	−0.199	0.010
1-oleoyl-sn-glycero-3-phosphoethanolamine	−0.502	0.189	−0.887	−0.118	0.012
Linoleoyl ethanolamide	−0.740	0.285	−1.319	−0.160	0.014
CP 47,497-C9-homolog	−0.628	0.245	−1.125	−0.130	0.015
Glycerol-3-phosphate	−0.753	0.296	−1.355	−0.152	0.015
(3alpha,5beta,7beta,17xi)-3,7,12-Trihydroxycholan-24-oic acid	−0.417	0.164	−0.753	−0.081	0.017
(9Z,12S,13R)-12,13-Dihydroxy-9-octadecenoic acid	−0.765	0.307	−1.388	−0.142	0.018
2-arachidonyl glyceryl ether	−0.372	0.149	−0.676	−0.068	0.018
Neohesperidin	−0.658	0.270	−1.207	−0.108	0.021
Thiamine	−0.694	0.286	−1.276	−0.113	0.021
pi-Methylimidazoleacetic acid	0.710	0.294	0.114	1.305	0.021
CP 47,497-C9-homolog	−0.714	0.301	−1.324	−0.104	0.023
17-Hydroxypregnenolone	−0.685	0.291	−1.275	−0.094	0.024
13-EPI-12-OXO PDA	0.708	0.301	0.096	1.320	0.025
Eicosapentaenoic acid methyl ester	−0.664	0.283	−1.240	−0.089	0.025
1-Methylproline	−0.671	0.290	−1.261	−0.081	0.027
Prostaglandin H1	0.726	0.317	0.084	1.368	0.028
Isodeoxycholic acid	−0.585	0.261	−1.116	−0.054	0.032
CP 47,497-C9-homolog	−0.508	0.242	−1.001	−0.016	0.043
NP-011548	−0.650	0.317	−1.294	−0.006	0.048
Palmitoyl ethanolamide	−0.631	0.310	−1.260	−0.002	0.049
3-linoleoyl-sn-glycerol	−0.640	0.321	−1.291	0.011	0.054
Estriol 17-sulfate	−0.538	0.275	−1.096	0.020	0.058
Lithocholic Acid	−0.618	0.317	−1.262	0.027	0.060
8-Hydroxy-2(1H)-quinolinone	−0.604	0.314	−1.241	0.032	0.062
5-[(1S,2R,4aR)-5-(Hydroxymethyl)-1,2,4a-trimethyl-1,2,3,4,4a,7,8,8a-octahydro-1-naphthalenyl]-3-methylpentanoic acid	−0.600	0.315	−1.238	0.038	0.065
(2R)-3-{[(2Aminoethoxy) (hydroxy)phosphoryl]oxy}-2-hydroxypropyl myristate	−0.515	0.273	−1.069	0.039	0.067
Alpha-Eleostearic acid	−0.346	0.186	−0.725	0.033	0.072
4-Pyridoxic acid	0.472	0.255	−0.049	0.992	0.074
D-(+)-Xylose	−0.542	0.297	−1.144	0.060	0.076
Stachydrine	−0.581	0.321	−1.231	0.069	0.078
Taurine	−0.589	0.326	−1.251	0.072	0.079
Bilirubin	−0.579	0.323	−1.235	0.076	0.082
Linoleoyl Ethanolamide	−0.425	0.239	−0.913	0.062	0.085
D-(+)-Glucose	−0.563	0.318	−1.207	0.082	0.085
(20R)-17-Hydroxy-3-oxopregn-4-en-20-yl hydrogen sulfate	0.485	0.275	−0.072	1.042	0.086
Conocarpan	0.510	0.290	−0.079	1.099	0.087
1,5-Isoquinolinediol	0.528	0.303	−0.089	1.145	0.091

Metabolites identify by linear regression model adjusted for recruitment cluster and treatment arm. Significance level set at *p* < 0.05.

## 4 Discussion

In this report, we leveraged a population of women from rural Guatemala where maternal micronutrient deficiencies and LBW are prominent (Lander et al., 2019). We show that (1) LBW was associated with specific maternal gut microbial taxa and modest changes in alpha diversity; (2) genus-level taxonomic data substantially improved accuracy of a machine learning model to predict LBW; and (3) fecal metabolites related to tryptophan metabolism, bile acids, fatty acids and endocannabinoid system were associated with LBW. These findings represent potential links between the maternal gut microbiome and host-microbial metabolite interactions during pregnancy and the risk of LBW infants.

At 12 weeks of gestation, mothers who delivered LBW infants showed increased abundance of several bacterial taxa known to be associated with pregnancy outcomes and growth. Higher *Sutterella* abundance during pregnancy has been associated with preterm birth (Ollberding et al., 2016) which may be related to its potential pro-inflammatory role (Ferrocino et al., 2018). Postnatally, *Sutterella* abundance has been found to be associated with rapid infant growth, fat mass, and weight loss (Gilley et al., 2022; Reyna et al., 2022; Peng et al., 2024) supporting a role for this taxon in growth. *Barnesiella*, a beneficial gut microbe and producer of the short chain fatty acid (SCFA) acetate, is known to increase throughout pregnancy and is associated with low-birth weight pups in mice (Wang et al., 2016). Another SCFA-producing bacterium elevated in the LBW group at 12 weeks, *Faecalibacterium,* was similarly higher in mothers who had fetal growth restricted infants (Tu et al., 2022) or premature labor (Mu et al., 2023). Lastly, *Fusobacterium,* a gram-negative bacterium associated with infections that is typically found in the oral cavity, gastrointestinal tract, and female genital tract (Chen et al., 2022), was increased during late pregnancy in the LBW group. Both oral and vaginal *Fusobacterium nucleatum* abundances are associated with pre-term birth/delivery (Cobb et al., 2017; Holst et al., 1994). Enrichment of *Fusobacterium* was identified in the fecal microbiome of pregnant mothers who gave birth prematurely (Yin et al., 2021), mothers with pre-eclampsia (Chen et al., 2020), and was associated with C-reactive protein levels in the blood of mothers with hyperglycemia (Gao et al., 2020). Published reports support our results that specific microbial species likely have a role in LBW. Further investigation is needed to identify the mechanistic connection between these bacterial taxa during pregnancy on LBW status.

Our results suggest a possible role of inflammation in the birth of LBW infants, which is in line with previous studies (Hunter et al., 2023). Multiple bacterial taxa altered in women who delivered LBW infants have known roles in regulating inflammation. As detailed above, *Sutterella* is known to be pro-inflammatory while *Faecalibacterium* has anti-inflammatory effects (Al Bander et al., 2020). The only genus increased among the LBW group at both pregnancy timepoints was *Bacteroides*, a gram-negative bacterium that contributes to lipopolysaccharide (LPS) biosynthesis. Increased *Bacteroides* abundance has been suggested to induce inflammation during pregnancy (Tang et al., 2023; Gorczyca et al., 2022) and abundance was higher in women with fetal growth restricted infants (Tu et al., 2022). *Flavonifractor*, thought to be an important taxon for establishing neonatal immunity, was higher in the LBW group at 34 weeks. Contrary to our results, lower abundance has been identified in LBW piglets (Li et al., 2018), in neonates from mothers with gestational diabetes (GDM; Soderborg et al., 2020), and in pregnant women with intrahepatic cholestasis (Zhan et al., 2021). This may be due to analysis conducted on infant fecal samples compared to pregnant mothers or different maternal factors (i.e., GDM or intrahepatic cholestasis) that could also impact the abundance of this bacterial taxon. Thus, more studies are needed to understand the role of *Flavonifractor* during pregnancy on fetal growth.

Several fecal metabolites with higher concentration associated with LBW also are linked to inflammation. Xanthurenic acid, a downstream metabolite of the kynureine pathway, was increased with LBW in our study, and in pregnant women with preeclampsia in work by others (van Zundert et al., 2022). Metabolites from the kynurenine pathway have been previously associated with inflammation, environmental enteric enteropathy, zinc homeostasis, birthweight and poor linear growth (Gazi et al., 2020; Kosek et al., 2016; Groer et al., 2018; Tan et al., 2022; Garay et al., 2022). Tang et al. also showed high rates of elevated intestinal and systemic inflammatory markers in the pregnant mothers from Gutamela compared to other sites of WF trial (Tang et al., 2022). In addition, bile acid metabolites (cholic acid, isodeoxycholic acid) also were associated with LBW in our study. Bile acids are thought to shape microbial composition via activation of host signaling pathways or antimicrobial effects on microbes (Ridlon and Gaskins, 2024). Dysregulation of bile acids in pregnant women can increase risk of preterm birth (You et al., 2020). Cholic acid in a normal pregnancy increases with progression of gestation (Gagnon et al., 2021). Cholic acid has been found be lower in feces from preterm infants (Kumagai et al., 2007) and negatively correlated with *Bacteroidetes*, *Staphylococcu*s and *Acinetobacter* in very low birth weight infants (Liu et al., 2023). At 34 weeks, we also showed an association of LBW with reduced *Turicibacter*, which is reported to modify host bile acids and lipid metabolism (Lynch et al., 2023). In settings of stress during pregnancy, similar lower relative abundance has been identified in pregnant women with HIV infection (Verdam et al., 2013), heat stressed pigs (Ridaura et al., 2013), and in calorie-restricted pregnant mice (Gilley et al., 2024). However, it was not found to be significantly altered in fecal samples from mothers with overweight/obesity during pregnancy (Ruebel et al., 2021).

Targeting pro-inflammatory gut microbes and metabolites may be a viable intervention to reduce incidence of LBW. In mice, the gut microbiome protected against fetal growth restriction through modulation of inflammatory pathways including toll-like receptors that recognize and respond to pro-inflammatory signals including LPS (Tang et al., 2023). Pregnant women with higher circulating levels of interleukin 17A and interleukin 1β were associated with increased risk of both preterm birth and LBW and associated with lower birth length z-score and lower weight for age z-scores (Shafiq et al., 2021). Another larger cohort study in healthy pregnant women, also found inflammatory related serum markers, C-reactive protein and vascular endothelial growth factor (VEGF)-D to be predictive of lower birthweight (Yeates et al., 2020). However, additional research is needed to thoroughly test this possibility.

Our machine learning model identified gestational length, maternal BMI, and relative abundances of *Oribacterium, Phascolarctobacterium, Roseburia, Bifidobacterium, Lachnospira, Fusobacterium,* and *Escherichia-Shigella* as contributors to predicting delivery of a LBW infant. The maternal variables shown to be predictive of LBW are in line with previous reports demonstrating associations with maternal height, weight, parity, SES, education, prenatal care, nutrition status and LBW status (Fedrick and Adelstein, 1978; Alsayeed et al., 2023; Jafree et al., 2015; Cluzeni et al., 2023). Of the microbial taxa identified, *Roseburia* was enriched at 12 weeks in mothers of LBW compared to NW infants, although it did not reach statistical significance During pregnancy, *Roseburia* is lower in women with gestational diabetes and obesity (Shen et al., 2024; Obuchowska et al., 2022). It has also been shown to be enriched in very low birth weight infants at 1 month of age (Chang et al., 2011), lower in infants with extrauterine growth restriction (Fan et al., 2021), and predictive of birthweight (Gough et al., 2021). These studies and our data show conflicting results, which may partially be due to the varying degree of growth restriction or birth weight between infants. However, this information can still highlight a potential role for *Roseburia* in predicting infant growth outcomes. Another taxon of note is *Fusobacterium,* which was enriched in the LBW group and mentioned above. In addition, both *Escherichia-Shigella* and *Phascolarctobacterium* were decreased in late pregnancy samples and *Lachnosipria* was increased in 12-week samples with LBW infants in this study and previously associated with low birth weight (Li et al., 2022) and fetal growth restriction (Zhang et al., 2019; Tang et al., 2024). Thus, these combined results suggest that not only maternal factors, but the maternal gut microbiome may be predictive of LBW status during pregnancy. However, additional more robust data sets will be needed to confirm this observation.

Our analysis also highlighted changes in fecal metabolites related to the endocannabinoid system that were associated with LBW. It is well known that the endocannabinoid system plays an important role in energy balance, metabolism, immune system and is a modulator of gut homeostasis and physiology (Ellermann, 2023; Drummen et al., 2020; Bambang et al., 2012). Closely related to leptin signaling, the endocannabinoid system, a lipid related signaling system, has emerged as a potential modulator of different biological processes involved in developmental programming (Keimpema et al., 2013). Recent evidence has suggested that altered nutrition during pregnancy may have an impact on the endocannabinoid system and lead to changes in brain function or behavioral development of offspring (Ramirez-Lopez et al., 2017; Matias et al., 2003; Ramirez-Lopez et al., 2016; Ramirez-Lopez et al., 2015). More specifically, maternal undernutrition is associated with lower levels of endocannonid related metabolites in different biological tissues (Matias et al., 2003; Ramirez-Lopez et al., 2016), mimicking a similar response to what we found in maternal stools samples. However, there is limited knowledge on the role of endocannabinoids and related compounds within the maternal-fetal dyads, although maternal endocannabinoids have been shown to be transferred by the placenta to the fetus (Keimpema et al., 2013; Chan et al., 2013; Kozakiewicz et al., 2021). As an example, cannabis use during pregnancy leads to increased risk of LBW infants, pre-term birth (Leemaqz et al., 2016), poor fetal growth (Conner et al., 2016), stillbirth (Varner et al., 2014) and other adverse neonatal outcomes (Kozakiewicz et al., 2021; Metz et al., 2017). In addition, specifically endocannabinoid related metabolites, plasma anandamide (AEA) and palmitylethanolamide (PEA) levels in pregnant women during late pregnancy predict pre-term birth (Bachkangi et al., 2019). Different endocannabinoid anandamides in the placenta were found to be associated with premature labor (Taylor et al., 2023). Combined with the growing knowledge that endocannabinoid and related metabolites may modulate the gut microbiome, specifically, microbial composition has also been shown to be associated with different endocannabinoid components, when altered by dietary patterns or antibiotics (Lacroix et al., 2019; Castonguay-Paradis et al., 2023; Guida et al., 2018; Tagliamonte et al., 2021). Our results highlight a potential system that could be influenced by gut microbiome during pregnancy and predict infant growth outcomes, such as LBW. Although more studies would be needed to understand this mechanism.

The present study is not without limitations. We did not consider the effect of pre-term infants, or exclude them from our analysis due to low sample number in the LBW group. On average in both 12-and 34-week samples there was a combined total of 5% of infants classified as preterm. Next, metabolomics analyses were conducted on a subset of 34-week samples, which limited incorporation of these data into predictive models. Future metabolomics studies with a larger sample set could provide insight into key metabolites that relate to bacterial abundances, and which predict LBW risk. We did not examine the direct impact of nutritional intervention treatment arms with LBW, but we did include this as a covariate for our analysis. Our previous publication, examining the gut microbiome from all four sites of the Women First Trial (Democratic Republic of the Congo, Guatemala, India, and Pakistan), found no association with supplement status and bacterial taxa abundance or alpha diversity metrics (Tang et al., 2022). In the present study, many of our differences were only nominally significant and did not pass multiple testing corrections, and hence should be interpreted with caution. Lastly, with the use of short-read 16S rRNA amplicon sequencing, we were unable to obtain species-level data, therefore, the present analyses reflect a broad view of the microbiome and leave room for future granular analyses.

In conclusion, our findings indicate that mothers who delivered LBW infants showed differences in their gut microbiome at both early and late pregnancy, compared to mothers who gave birth to NW infants. Maternal factors such as BMI, gestational length, and individual bacterial taxa predict LBW status. We also showed associations of LBW with maternal fecal metabolites related to tryptophan metabolism, endocannabinoid system, fatty acids, and bile acids. Combined with findings from others, the results presented here may suggest a functional role of the maternal microbiome in mediating adverse growth outcomes in offspring. In addition, the maternal gut microbiome and metabolites may be potential biomarkers that could be used to predict LBW infants or targets for future interventions to reduce prevalence of LBW.

## Data Availability

This study is available at the NIH Common Fund’s National Metabolomics Data Repository (NMDR) website, the Metabolomics Workbench, https://www.metabolomicsworkbench.org where it has been assigned Study ID ST003508. The data can be accessed directly via its Project DOI: http://dx.doi.org/10.21228/M8681W.

## Glossary

LBW: Low birth weight
NW: Normal birth weight
WF: The women first: preconception maternal intervention nutrition trial
sqLNS: Small-quantity lipid-based micronutrient supplement
QC: Quality control
SDMO: Sulfadimethoxine
HESI: Heat-electrospray ionization
GLM: Generalized linear model
VIP: Variable importance
SGA: Small for gestational age
WAZ: Weight-for-age z-score
LAZ: Length-for-age z-score
HCAZ: Head circumference-for-age z-score
NMDS: Non-metric dimensional scaling
PCA: Principal Component Analysis
PLDS-DA: Partial least squares discriminant analysis.
BMI: Body mass index
SCFA: Short chain fatty acid
LPS: Lipopolysaccharide
GDM: Gestational diabetes mellitus
VEGF-D: Vascular endothelial growth factor-D
AEA: Anandamide
PEA: Palmitylethanolamide
