# Supplementary material for: Associations between maternal microbiome, metabolome and incidence of low-birth weight in Guatemalan participants from the Women First Trial
Source: Front Microbiol. 2024 Oct 15;15:1456087. doi: 10.3389/fmicb.2024.1456087 (PMC11518777; doi:10.3389/fmicb.2024.1456087)
Supplement: Supplementary file 1 [file Table_1.DOCX]

**SUPPLEMENTARY METHODS**

*Chromatography and Mass Spectrometry*

An Orbitrap Exploris 480 mass spectrometer (ThermoFisher Scientific, Waltham, MA) interfaced with the Vanquish UHPLC system and fitted with high flow and low flow heat-electrospray ionization (HESI) probes was used for instrumental analysis. A flow rate of 0.3 mL min–1 was employed to carry out chromatographic separations using an Acquity Premier CSH C18 1.7 μm × 2.1 × 100 mm Column (Waters, USA). Water (A) and acetonitrile (B), both acidified with 0.1% formic acid, made up the mobile phase system. The LC gradient was set to 0 min, 0% B; 2 min, 40% B; 8 min, 98% B; 10 min, 98% B; 10.5 min, 0% B; 15 min, 0% B. Throughout the analysis, a 40 °C column temperature and a 5.0 μL injection volume were employed.

The samples were analyzed in both positive and negative ionization mode. The electrospray ionization voltage was kept at 3.6 kV in the positive mode and 3.5 kV in the negative mode. Sheath gas was set to 35, auxiliary gas was set at 10, and sweep gas was set to 1 arbitrary unit (Arb). The ion transfer tube temperature (ITT) was set at 350°C and the vaporizer temperature was set at 350°C. The scan parameters for the mass spectrometer included 6 s chromatogram peak width and a 15-min duration time in DDA mode. Full MS1 used the Orbitrap mass analyzer with a resolution of 180,000, scan range (m/z) of 60–900 in positive mode and 58-870 in negative mode, maximum injection time (MIT) of 100, automatic gain control (AGC) target of 5e5, 1 microscan, and RF lens set to 70. MS/MS analysis was performed on the pooled QC samples using six rounds of iterative DDA (ThermoFisher AcquireX) at a resolution of 30,000 FWH using a stepped HCD collision energies of 20, 40, and 60 V. Other MS/MS parameters included: MIT of 50 ms, microscan set to 1, AGC set to 1e6, and a scan range of 60–900 in positive mode and 58-870 in negative mode. The top 5 abundant precursors within an isolation window of 2 m/z were chosen for MS/MS analysis. Xcalibur v4.4.16.14 (ThermoFisher Scientific, Waltham, MA) was used for instrument control and data acquisition.

*Data Analysis*

Chromatograms of the spiked reference standards were viewed and processed using Free Style 1.8 software (ThermoFisher Scientific, Waltham, MA). The ICIS algorithm, which is one of the functions in the Free Style 1.8 software, was used for peak detection. Compound Discoverer (v3.3, ThermoFisher Scientific, Waltham) was used to predict chemical formulas, align retention times across samples, remove background noises, group compounds, and explore spectral (MS1 and MS2) annotation sources and assigns compound annotations using a node-based methodology (Figure 2). Briefly, the ChromAlign model was employed to align retention time to a reference QC file. The detect compounds node was used to implement peak detection for unknowns using a 50,000 as a minimum peak intensity, 30% intensity tolerance, 5 ppm mass tolerance and a 3 signal to noise threshold for extracted ion chromatograms (EIC). All detected compounds were grouped across samples using the group compounds node with 5 ppm mass error and 0.2 min retention time shift for the preferred ions [M+H]+1 or [M-H]-1. The predict compositions node was used to predict the chemical formulas of the unknown compounds by setting the mass tolerance for the EICs at 5 ppm and intensity tolerance of 30% for isotope pattern matching. The mzCloud data base was searched for matching fragmentation spectra using the search mzCloud node. The data base was searched for all classes of compounds using 10 ppm precursor mass tolerance, 10 ppm fragment mass tolerance, Cosine algorithm for identity search and 20 as activation energy tolerance. Search mzVault node was employed to search for an in-house spectral data base matching (spectral library for 430 metabolites) as well as local NIST data base search for compounds of interest. The search ChemSpider against the Kyoto Encyclopedia of Genes and Genomes (KEGG), human metabolome database, and BioCyc was used search mass spectral databases for matching compounds by formula or mass within 5 ppm mass tolerance. Chemspider results were ranked by searching for similarity for all compounds with ddMS2 using the mzCloud and mzLogic algorithms. The search mass list node enabled the search for masses (within 5 ppm) and retention time (RT, 0.6 min tolerance) that matched the detected compounds using local mass list containing RT information for 340 compounds. Background compounds were defined as those with a sample to blank abundance ratio of 5 or below. Chromatographic peaks that were detected by the detect Compounds node in one of the input files but were missing from other input files in the file set were redetected using the peak detection algorithm in the fill gaps node. The Apply SERRF QC correction node was used for batch effect normalization using systematic error removal with random forests (SERRF) algorithm. This normalization was applied for the compounds that were detected in at least 50% of the QC samples within maximum of QC area relative standard deviation (RSD) of 30%. Annotation sources were prioritized using the Assign Compound annotation node. The Full MS data was used for peak picking and the ddMS/MS data for identification only. Following data processing, features were excluded using general filters such as background removal, mass accuracy (delta = ± 0.5 ppm), %RSD in QC, and peak quality. Compounds were then further filtered based on their level of confidence of identifications. Compounds with MS/MS spectrum match in either the in-house spectral library, mzCloud spectral data base or the local NIST spectral data base were putatively annotated as level 2. Compounds that showed a molecular formula or mass match in the ChemSpider data base was grouped as level 3 confidence of annotations. Features with only molecular formulas and/or accurate masses were not assigned any level.


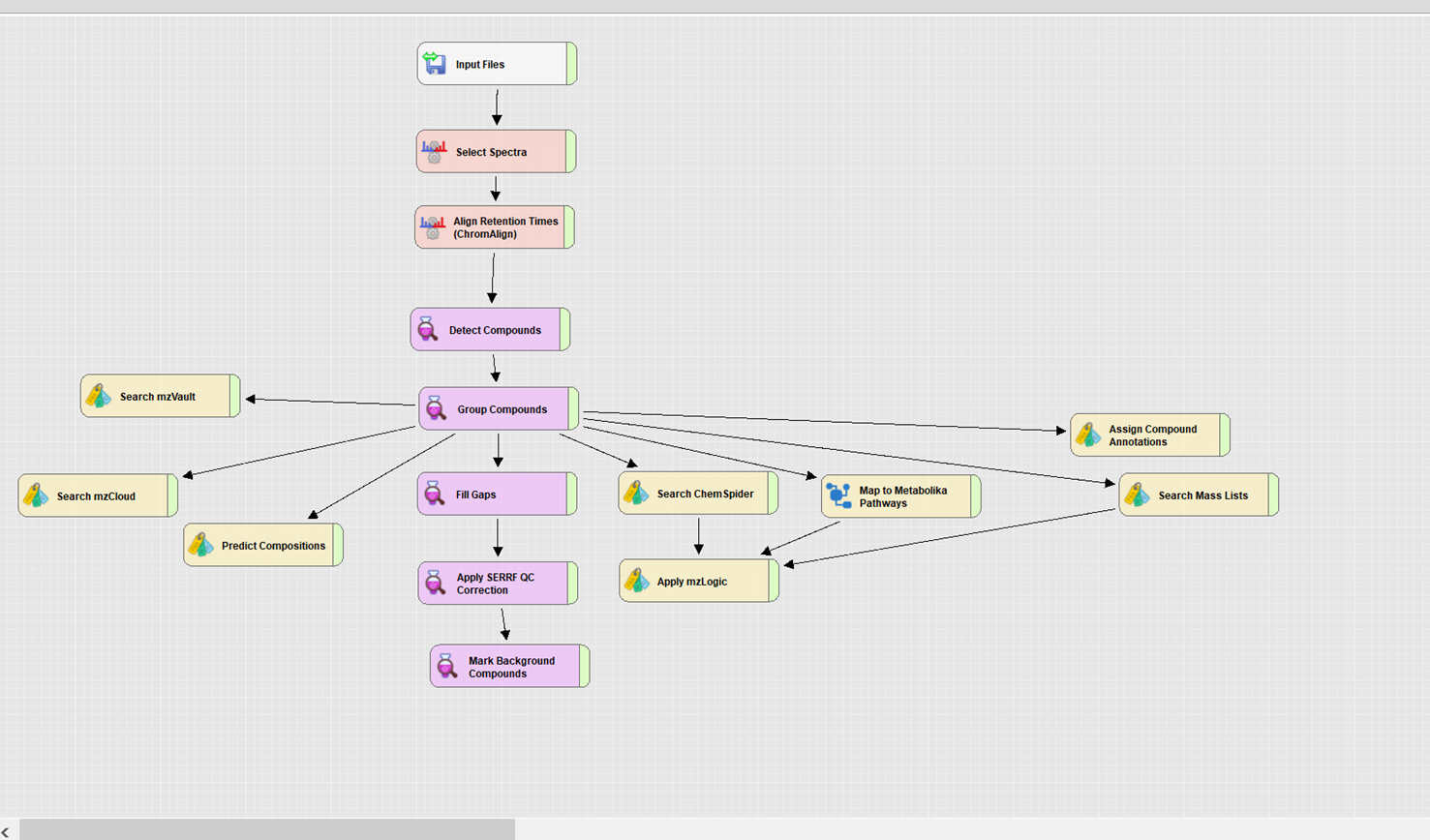


Supplementary Figure 1: Workflow tree from Compound Discoverer 3.3.0 software displaying data processing nodes and the associated workflow connection
